# Genetic polymorphisms analysis of pharmacogenomic VIP variants in Bai ethnic group from China

**DOI:** 10.1002/mgg3.884

**Published:** 2019-07-30

**Authors:** Wanlu Chen, Heng Ding, Yujing Cheng, Qi Li, Run Dai, Xin Yang, Chan Zhang

**Affiliations:** ^1^ Department of Blood Transfusion The First People's Hospital of Yunnan Province Kunming Yunnan Province China; ^2^ Honghe Center Blood Station Mengzi Yunnan Province China

**Keywords:** bai ethnic group, genetic polymorphisms, individualized medicine, pharmacogenomics, VIP variants

## Abstract

**Background:**

The pharmacogenomics study has been widely used for the study of very important pharmacogenetic (VIP) variants among different ethnic groups. However, there is little known about the pharmacogenomics information regarding Bai family. Our study aimed to screen the polymorphism of the VIP gene in Bai nationality.

**Methods:**

We genotyped 81 VIP variants (selected from the PharmGKB database) in the Bai population and then compared them to the other 11 major HapMap populations by chi‐square test, structure and F‐statistics (Fst) analysis.

**Results:**

Our results indicated that rs20417 (*PTGS2*), rs4148323 (*UGT1A*), and rs1131596 (*SLC19A1*) were most different in Bai compared with most of the 11 populations from the HapMap data set. Furthermore, population structure and F‐statistics (Fst) analysis also demonstrated that the Bai population has the closest genetic relationship with Han Chinese in Beijing, China (CHB), followed by Japanese in Tokyo, Japan (JPT), and the farthest population from the Yoruba in Ibadan, Nigeria (YRI).

**Conclusions:**

Our study not only presented the genotype frequency difference between the selected population of the Bai population and the other 11 populations, but also showed that the Bai population is most similar to the CHB populations, followed by JPT. These findings would contribute to the development of individualized medicine for the Bai population.

## 1 BACKGROUND

Personalized medicine, also known as precision medicine, refers to a customized medical model based on personal genomic information to design the best treatment for patients to achieve maximum therapeutic effect and minimize side effects (Jorgensen, 2015). Pharmacogenomics is an aspect of personalized medicine that is used to explore the impact of genetic variation on drug response (Yunus et al., 2013). Currently, most pharmacogenomics studies are focused on very important drug genes (VIPs) thought to be involved in the pharmacokinetics or pharmacodynamics of clinically relevant drugs (Jin, Shi, et al., 2016). These VIP genes play a crucial role in drug metabolism, transport, efficacy or drug response processes, and have been summarized in the pharmacogenetics and pharmaco‐genomics knowledge base in detail.

At present, the Pharmacogenetics and Pharmacogenomics Knowledge Base (PharmGKB: http://www.pharmgkb.org) is the most comprehensive database, which have collected amounts of genotype and phenotypic information related to the pharmacogenomic genome and systematically classify for these information (Jin, Zhao, et al., 2016). It is dedicated to reveal the relationship between these genetic variants and drug responses, and then providing patients with the most appropriate drug type and accurate guidance for optimal doses, so as to improve the drug efficacy and safety (Zhang et al., 2014). The PharmGKB currently contains information on more than 4,654 drugs, 4,067 diseases and 27,007 genotypic variants, and its knowledge delivers in a variety of forms, including VIP summaries, drug pathway diagrams, and curated literature annotations (Jin, Xun, et al., 2015).

As we all know, there are 56 ethnic groups in China. The Bai nationality is one of the fifteen ethnic minorities in China, with a long history in the region and distinct culture and traditions. The 861,895 people (According to the 2010 census) of the Bai ethnic minority reside primarily in Dali Bai Autonomous Prefecture of Yunnan Province (Pei, Zhengwei, Yijuan, Chai, & Zhang, 2017). Additionally, there are also distributions in Sichuan and Chongqing provinces. In recent years, pharmacogenomics research on genotypes and drug metabolism among different ethnic groups has become more common (Jin, Aikemu, et al., 2015), For example, several articles have reported that individuals carrying CYP2C9^*^2 and ^*^ 3 allele variants have lower dose requirements or warfarin sensitivity in Brazilian populations (Fohner, Brackman, Giacomini, Altman, & Klein, 2017). Shi et al. researched the genetic polymorphism of VIP variation in the pharmacogenomic of the Himalayan Deng people in southeast Tibet (Shi et al., 2015). Li et al. investigated the genetic polymorphism of very important pharmacogenomic variants in the Zhuang ethnic group of Southwestern China (Li et al., 2018). However, no report has addressed studies of pharmacogenomics information regarding the Bai nationality. Here, we are the first to propose a systematic research on the genetic polymorphism of VIP variants in the Bai ethnic group, which is of great significance for understanding the Bai population and further helping to diagnose, prevent and treat specific diseases.

In this study, we randomly selected and genotyped 81 VIP variants from the PharmGKB database in 100 Dali Bai Autonomous Prefecture from Yunnan province, aimed to identify the allele frequencies of VIP variants in the Bai nationality and to determine the difference in allele frequencies between the Bai nationality and 11 populations from the HapMap data set. The results could not only expand our understanding of ethnic diversity and pharmacogenomics, but also provide a solid theoretical basis for safer administration of drugs and better individualized treatments among the Bai population.

## 2 METHODS

### 2.1 Ethics statement

All volunteers were informed about the procedures and purpose of the study, both orally and in writing. They also agreed to provide blood samples and signed informed consent forms. The clinical protocol was approved by the Ethics Committee of Yunnan First People's Hospital and was performed in accordance with the Declaration of Helsinki.

### 2.2 Study participants

According to detailed recruitment and exclusion criteria, we recruited a random sample of 200 healthy, unrelated white people (100 men and 100 women) from Dali Bai Autonomous Prefecture in China's Yunnan province between July and October 2017. The incorporation criteria for all participants were as follows: (a) All individuals had exclusive Bai ancestry for at least the past three generations; (b) There was no genetic relationship among all participants; (c) All subjects were confirmed to be in good health through a routine medical history and physical examination and had no hereditary disease.

### 2.3 VIP loci selection and genotyping

We searched the PharmGKB database (https://www.pharmgkb.org/) and 81 random VIP variants of 40 genes were ultimately selected for our study according to available data on frequency, functionality, and linkage based on published research. The minor allele frequency of these SNPs sites in Chinese Han population was >0.05, which increase the statistical efficacy. According to the manufacturer's protocols, the genomic DNA was extracted from peripheral blood (5 ml) using a GoldMag‐ Mini Whole Blood Genomic DNA Purification Kit (GoldMag Co. Ltd., Xi'an, China). Then we measured the DNA concentration and purity with the NanoDrop spectrophotometer 2000 C (Thermo Scientific, Waltham, Massachusetts, USA). We designed polymerase chain reaction extended primers for these SNPS using the MassARRAY Assay Design 3.0 software. Agena MassARRAY RS1000 instrument (Shanghai, China) system was used for SNP genotyping analysis according to the standard scheme recommended by the manufacturer (Gabriel, Ziaugra, & Tabbaa, 2009). SNPs genotyping data were managed and analyzed by Agena Typer 4.0 software.

### 2.4 HapMap genotype data

The genotype data of the 11 populations were downloaded from the International HapMap Project web site (HapMap release127) at http://hapmap.ncbi.nlm.nih.gov.

The 11 populations are as follows: (a) African ancestry in Southwest USA (ASW); (b) Utah, USA residents with Northern and Western European ancestry from the CEPH collection (CEU); (c) Han Chinese in Beijing, China (CHB); (d) Punjabi in Lahore, Pakistan (PJL); (e) Gujarati Indians in Houston, Texas, USA (GIH); (f) Japanese in Tokyo, Japan (JPT); (g) Luhya in Webuye, Kenya (LWK);(h)Mexican Ancestry in Los Angeles, Colombia (MXL); (i) Peruvian in Lima, Peru (PEL); (j) Toscani in Italy (TSI); and (h) Yoruba in Ibadan, Nigeria (YRI).

### 2.5 Statistical analysis

We performed data processing and statistical analysis using Microsoft Excel (Redmond, WA, USA) and SPSS 17.0 statistical software package (SPSS, Chicago, IL, USA), including Hardy‐Weinberg equilibrium (HWE) analysis and *χ* ^2^ test. Accurate testing was used to determine whether the genotype frequency of each VIP variant in the Bai populations deviated from the HWE balance. The genotype frequencies of the Bai and 11 HapMap populations were calculated and compared using the *χ* ^2^ test. All *p* values were obtained two‐sided, and *p *< .05 were considered statistically significant before correction. In order to reduce the error detection rate of multiple tests, after Bonferroni correction, *p *< .05/(81*11) was indicated statistically significant. The structure (version 2.3.4) software (Excoffier, Laval, & Schneider, 2007) was used to analyze and compare the genetic structure of 12 populations. The value of Fst was calculated using Arlequin (version 3.1) software to infer the degree of genetic differentiation between populations (Evanno, Regnaut, & Goudet, 2005).

## 3 RESULTS

### 3.1 VIP variants identification

By searching the VIP variants listed in the PharmGKB database, we selected 81 VIP variants for study. The basic characteristics of the selected variants were listed in Table 1, including SNP, gene name, chromosome number and location, corresponding protein function, allelic variation, and genotype frequency.

**Table 1:** Basic Characteristics of the selected VIP variants from the PharmGKB database and genotype frequencies in Bai population

SNP ID	Genes	Chr	Position	Functional consequence	Allele		Allele frequencies	
					A	B	A (%)	B (%)
rs1801131	*MTHFR*	1	11,794,419	Missense	G	T	0.25	0.75
rs1801133	*MTHFR*	1	11,796,321	Missense	A	G	0.25	0.75
rs890293	*CYP2J2*	1	59,926,822	Upstream variant 2KB	A	C	0.08	0.92
rs3918290	*DPYD*	1	97,450,058	Splice donor variant	T	C	0.00	1.00
rs1801159	*DPYD*	1	97,515,839	Intron variant, missense	C	T	0.18	0.82
rs1801265	*DPYD*	1	97,883,329	Intron variant, missense, nc transcript variant, utr variant 5 prime	G	A	0.26	0.74
rs6025	*F5*	1	169,549,811	Missense	T	C	0.01	0.99
rs5275	*PTGS2*	1	186,673,926	Utr variant 3 prime	G	A	0.40	0.60
rs20417	*PTGS2*	1	186,681,189	Nc transcript variant, upstream variant 2KB	G	C	0.20	0.80
rs689466	*PTGS2*	1	186,681,619	Downstream variant 500B, upstream variant 2KB	C	T	0.22	0.78
rs4124874	*UGT1A1*	2	233,757,013	Intron variant, upstream variant 2KB	G	T	0.42	0.58
rs10929302	*UGT1A1*	2	233,757,136	Intron variant, upstream variant 2KB	A	G	0.30	0.70
rs4148323	*UGT1A1*	2	233,760,498	Intron variant, missense	A	G	0.03	0.97
rs1805124	*SCN5A*	3	38,603,929	Missense	C	T	0.23	0.77
rs6791924	*SCN5A*	3	38,633,208	Missense	A	G	0.04	0.96
rs3814055	*NR1I2*	3	119,781,188	Upstream variant 2KB, utr variant 5 prime	T	C	0.32	0.68
rs2046934	*P2RY12*	3	151,339,854	Intron variant	G	A	0.13	0.87
rs1065776	*P2RY1*	3	152,835,839	Synonymous codon	T	C	0.11	0.89
rs701265	*P2RY1*	3	152,836,568	Synonymous codon	G	A	0.37	0.63
rs975833	*ADH1A*	4	99,280,582	Intron variant	G	C	0.40	0.60
rs2066702	*ADH1B*	4	99,307,860	Missense	A	G	0.05	0.95
rs698	*ADH1C*	4	99,339,632	Missense, nc transcript variant	C	T	0.21	0.79
rs17244841	*HMGCR*	5	75,347,030	Intron variant	A	T	0.04	0.96
rs3846662	*HMGCR*	5	75,355,259	Intron variant	G	A	0.38	0.62
rs1042713	*ADRB2*	5	148,826,877	Missense	G	A	0.48	0.52
rs1042714	*ADRB2*	5	148,826,910	Missense	G	C	0.20	0.80
rs1142345	*TPMT*	6	18,130,687	Missense	T	C	0.04	0.96
rs2066853	*AHR*	7	17,339,486	Missense	A	G	0.27	0.73
rs1045642	*ABCB1*	7	87,509,329	Synonymous codon	A	G	0.40	0.60
rs1128503	*ABCB1*	7	87,550,285	Synonymous codon	G	A	0.42	0.58
rs2740574	*CYP3A4*	7	99,784,473	Upstream variant 2KB	C	T	0.23	0.77
rs3807375	*KCNH2*	7	150,970,122	Intron variant	C	T	0.43	0.57
rs4646244	*NAT2*	8	18,390,208	Intron variant, upstream variant 2KB	A	T	0.26	0.74
rs4271002	*NAT2*	8	18,390,758	Intron variant, upstream variant 2KB	C	G	0.14	0.86
rs1801280	*NAT2*	8	18,400,344	Missense	C	T	0.29	0.71
rs1799929	*NAT2*	8	18,400,484	Synonymous codon	T	C	0.27	0.73
rs1208	*NAT2*	8	18,400,806	Missense	G	A	0.32	0.68
rs1799931	*NAT2*	8	18,400,860	Missense	A	G	0.08	0.92
rs12248560	*CYP2C19*	10	94,761,900	Upstream variant 2KB	T	C	0.15	0.85
rs4986893	*CYP2C19*	10	94,780,653	Stop gained	A	G	0.01	0.99
rs4244285	*CYP2C19*	10	94,781,859	Synonymous codon	A	G	0.22	0.78
rs1057910	*CYP2C9*	10	94,981,296	Missense	C	A	0.05	0.95
rs7909236	*CYP2C8*	10	95,069,673	Upstream variant 2KB	T	G	0.14	0.86
rs17110453	*CYP2C8*	10	95,069,772	Upstream variant 2KB	C	A	0.17	0.83
rs2070676	*CYP2E1*	10	133,537,633	Intron variant	G	C	0.31	0.69
rs1695	*GSTP1*	11	67,585,218	Missense	G	A	0.35	0.65
rs1138272	*GSTP1*	11	67,586,108	Missense	T	C	0.03	0.97
rs1800497	*ANKK1*	11	113,400,106	Missense	A	G	0.32	0.68
rs6277	*DRD2*	11	113,412,737	Synonymous codon	A	G	0.24	0.76
rs1801028	*DRD2*	11	113,412,762	Missense	C	G	0.03	0.97
rs4149015	*SLCO1B1*	12	21,130,388	Upstream variant 2KB	A	G	0.05	0.95
rs2306283	*SLCO1B1*	12	21,176,804	Missense	A	G	0.38	0.62
rs4149056	*SLCO1B1*	12	21,178,615	Missense	C	T	0.09	0.91
rs731236	*VDR*	12	47,844,974	Synonymous codon	G	A	0.28	0.72
rs7975232	*VDR*	12	47,845,054	Intron variant	A	C	0.48	0.52
rs1544410	*VDR*	12	47,846,052	Intron variant	T	C	0.30	0.70
rs2239185	*VDR*	12	47,850,776	Intron variant	A	G	0.50	0.50
rs1540339	*VDR*	12	47,863,543	Intron variant	C	T	0.39	0.61
rs2239179	*VDR*	12	47,863,983	Intron variant	C	T	0.36	0.64
rs3782905	*VDR*	12	47,872,384	Intron variant	C	G	0.24	0.76
rs4516035	*VDR*	12	47,906,043	Upstream variant 2KB	C	T	0.18	0.82
rs11568820	*None*	12	47,908,762	None	T	C	0.46	0.54
rs762551	*CYP1A2*	15	74,749,576	Intron variant	C	A	0.37	0.63
rs3760091	*SULT1A1*	16	28,609,479	Intron variant, upstream variant 2KB	G	C	0.36	0.64
rs7294	*VKORC1*	16	31,091,000	Upstream variant 2KB, utr variant 3 prime	T	C	0.42	0.58
rs9934438	*VKORC1*	16	31,093,557	Intron variant	G	A	0.36	0.64
rs1800566	*NQO1*	16	69,711,242	Missense	A	G	0.29	0.71
rs2108622	*CYP4F2*	19	15,879,621	Missense	T	C	0.24	0.76
rs8192726	*CYP2A6*	19	40,848,591	Intron variant	A	C	0.10	0.90
rs1801272	*CYP2A6*	19	40,848,628	Missense	T	A	0.01	0.99
rs28399433	*CYP2A6*	19	40,850,474	Upstream variant 2KB	C	A	0.13	0.87
rs3211371	*CYP2B6*	19	41,016,810	Downstream variant 500B, missense, utr variant 3 prime	T	C	0.05	0.95
rs5629	*PTGIS*	20	49,513,169	Stop gained, synonymous codon	T	G	0.22	0.78
rs1051298	*SLC19A1*	21	45,514,912	Intron variant, utr variant 3 prime	A	G	0.48	0.52
rs1051296	*SLC19A1*	21	45,514,947	Intron variant, utr variant 3 prime	C	A	0.49	0.51
rs1051266	*SLC19A1*	21	45,537,880	Missense, utr variant 5 prime	T	C	0.49	0.51
rs1131596	*SLC19A1*	21	45,538,002	Synonymous codon, utr variant 5 prime	A	G	0.48	0.52
rs4680	*COMT*	22	19,963,748	Missense, upstream variant 2KB	A	G	0.37	0.63
rs59421388	*CYP2D6*	22	42,127,608	Missense, synonymous codon, upstream variant 2KB	T	C	0.03	0.97
rs28371725	*CYP2D6*	22	42,127,803	Intron variant, upstream variant 2KB	T	C	0.06	0.94
rs61736512	*CYP2D6*	22	42,129,132	Intron variant, missense, upstream variant 2KB	T	C	0.03	0.97

Note

SNP, Single nucleotide polymorphism; Chr, Chromosome; A, reference allele; B, other allele.

### 3.2 Statistical analyses of 81 loci among 11 populations

Compared the genotype frequency distribution between the Bai population and these 11 HapMap populations by *χ* ^2^ test combined with Bonferroni correction multiple hypotheses and multiple comparisons [*p* < .05/(81^*^11)]. The genotype frequencies of 81 loci in HapMap 11 populations were listed in Table 2. Without Bonferroni's correction (*p* < .05), there were 45, 51, 8, 49, 53, 18, 55, 40, 50, 51, and 50 variants that differed in frequency in the Bai population compared to the ASW, CEU, CHB, PJL, GIH, JPT, LWK, MXL, PEL, TSI, and YRI populations, respectively. After adjustment, the number of VIP variants has updated and were recorded as follows: 32, 35, 3, 35, 35, 3, 40, 24, 24, 35, and 43, which corresponds to the order illustrated before. However, compared with Bai, the YRI population contained the most different VIP variants loci after Bonferroni adjustment, indicating that YRI was the most different race from Bai. At the same time, compared with the other 11 populations, the rs20417 (*PTGS2*: OMIM: 600262), rs4148323 (*UGT1A*: OMIM: 191740), and rs1131596 (*SLC19A1*: 600424) locus presented the greatest number of significant differences in the Bai ethnic population. While the rs890293 (*DPYD*: OMIM: 612779), rs3918290 (*DPYD*), rs6025 (*F5*: OMIM: 612309), rs2046934 (*P2RY12*: OMIM: 600515), rs4646244 (*NAT2*: OMIM: 608490), rs4986893 (*CYP2C19*: OMIM:124020), rs1057910 (*CYP2C9*: OMIM: 601130), rs1800497 (*ANKK1*: OMIM: 608774), rs8192726 (*CYP2A6*: OMIM: 12270), rs5629 (*PTGIS*: OMIM: 601699), rs1051298 (*SLC19A1*), rs1051296S (*LC19A1*) has no significant genetic differences between Bai nationality and the 11 HapMap populations. The genotype counts of 81 loci in 11 HapMap populations listed in Table S1.

**Table 2:** Bai compared with 11 HapMap populations after Bonferroni's multiple adjustments

Gene	SNP ID	*p* < .05/(81*11)											Different Populations
		ASW	CEU	CHB	PJL	GIH	JPT	LWK	MXL	PEL	TSI	YRI	
*MTHFR*	rs1801131	—	7.05E−04	—	**3.60E−08**	**2.00E−08**	—	—	—	1.29E−02	2.04E−03	—	2
	rs1801133	**2.70E−07**	—	—	**1.10E−08**	**3.20E−08**	—	**3.20E−14**	—	—	—	**9.90E−12**	5
*CYP2J2*	rs890293	—	—	2.06E−02	—	—	1.75E−03	—	3.43E−02	1.87E−04	—	—	0
*DPYD*	rs3918290	—	—	—	—	—	—	—	—	—	—	—	0
	rs1801159	—	**4.51E−02**	—	**2.30E−05**	1.06E−04	—	—	—	**3.60E−08**	3.98E−02	1.57E−02	2
	rs1801265	**5.70E−18**	—	—	**4.20E−08**	**3.90E−11**	—	**1.40E−21**	**7.40E−05**	—	2.70E−04	**4.90E−19**	5
*F5*	rs6025	—	1.67E−02	—	—	—	—	—	—	—	—	—	0
*PTGS2*	rs5275	**2.50E−15**	**1.02E−07**	—	**1.50E−11**	**1.90E−07**	4.47E−02	**2.90E−22**	1.19E−04	**5.60E−08**	1.46E−03	**3.50E−27**	7
	rs20417	**1.30E−26**	**3.16E−15**	**1.20E−05**	**1.10E−19**	**6.40E−16**	1.06E−04	**2.40E−24**	**9.80E−20**	**4.80E−17**	**1.40E−16**	**5.50E−33**	10
	rs689466	**7.20E−08**	**2.04E−07**	—	**8.40E−11**	**1.50E−11**	—	**5.60E−21**	4.95E−03	1.39E−03	**2.80E−07**	**9.70E−17**	7
*UGT1A1*	rs4124874	**3.60E−21**	1.14E−03	—	**6.50E−16**	**1.10E−12**	—	**1.80E−38**	**2.80E−06**	**5.60E−12**	6.79E−04	**4.70E−42**	7
	rs10929302	**3.30E−14**	**3.81E−13**	—	**1.30E−21**	**8.50E−21**	1.50E−04	**2.10E−17**	**1.10E−13**	**7.50E−23**	**4.80E−08**	**1.00E−16**	9
	rs4148323	**3.00E−11**	**1.29E−17**	4.04E−02	**9.20E−17**	**2.10E−15**	**1.50E−05**	**1.30E−17**	**2.40E−10**	**1.30E−15**	**9.80E−19**	**7.10E−19**	10
*SCN5A*	rs1805124	1.65E−02	—	—	1.91E−02	—	—	3.52E−04	—	4.78E−02	—	**1.70E−05**	1
	rs6791924	—	—	—	—	—	—	**8.20E−15**	—	—	—	**2.10E−08**	2
*NR1I2*	rs3814055	—	2.81E−03	—	2.17E−02	**7.30E−07**	—	2.83E−02	9.86E−03	**5.40E−05**	3.15E−04	—	2
*P2RY12*	rs2046934	—	—	—	—	3.27E−02	‐	3.49E−02	—	4.00E−03	—	—	0
*P2RY1*	rs1065776	**1.70E−05**	—	—	2.10E−03	—	—	**6.30E−07**	—	—	2.90E−02	**2.00E−07**	3
	rs701265	**7.00E−12**	7.34E−04	—	—	2.54E−02	—	**5.80E−26**	—	—	2.59E−04	**2.30E−27**	3
*ADH1A*	rs975833	**6.80E−22**	**5.66E−28**	—	**1.20E−14**	**1.20E−10**	—	**1.70E−32**	**2.40E−32**	**1.50E−38**	**1.00E−26**	**2.20E−24**	9
*ADH1B*	rs2066702	**7.90E−18**	—	—	—	—	—	—	—	—	—	**1.20E−27**	2
*ADH1C*	rs698	—	**5.61E−23**	—	**3.00E−11**	**2.40E−08**	—	—	**2.90E−07**	1.11E−02	**2.30E−10**	—	5
*HMGCR*	rs17244841	—	—	—	—	—	—	**3.80E−08**	—	—	3.28E−03	**7.20E−09**	2
	rs3846662	**1.20E−15**	—	—	6.40E−03	**1.70E−06**	2.12E−02	**3.10E−32**	—	2.39E−02	—	**3.70E−32**	4
*ADRB2*	rs1042713	—	**3.79E−05**	—	4.53E−02	2.26E−02	—	—	—	1.30E−02	1.15E−04	—	1
	rs1042714	—	**2.11E−19**	—	7.30E−05	7.10E−05	—	3.32E−04	—	—	**5.70E−16**	—	2
*TPMT*	rs1142345	—	—	—	—	—	—	**1.60E−08**	—	—	—	2.46E−04	1
*AHR*	rs2066853	—	**3.92E−10**	—	**3.40E−06**	**7.50E−09**	3.49E−02	5.83E−03	**2.60E−05**	9.92E−03	**4.30E−10**	—	5
*ABCB1*	rs1045642	**5.90E−07**	1.06E−02	—	—	6.02E−03	—	**2.00E−12**	—	—	—	**3.30E−15**	3
	rs1128503	**5.20E−23**	**2.37E−10**	—	1.21E−04	1.08E−03	1.90E−02	**2.60E−35**	**3.80E−06**	**2.10E−15**	**8.70E−11**	**4.70E−33**	7
*CYP3A4*	rs2740574	**1.70E−51**	—	—	—	—	—	**6.10E−60**	—	—	—	**4.70E−62**	3
*KCNH2*	rs3807375	—	**4.29E−22**	—	**1.50E−18**	**1.70E−19**	—	—	**4.40E−06**	—	**3.80E−24**	—	5
*NAT2*	rs4646244	—	—	—	4.19E−02	2.37E−03	—	—	1.86E−02	1.58E−04	—	—	0
	rs4271002	3.38E−02	**3.49E−05**	—	—	4.73E−02	—	3.27E−04	—	3.58E−02	—	**9.70E−05**	1
	rs1801280	**2.50E−16**	**4.31E−28**	—	**3.90E−28**	**3.80E−21**	—	**1.60E−24**	**3.20E−21**	**1.80E−17**	**3.10E−29**	**1.70E−13**	9
	rs1799929	**4.50E−13**	**5.20E−28**	—	**2.30E−25**	**1.20E−18**	—	**2.10E−21**	**2.00E−20**	**8.80E−17**	**3.10E−29**	**8.90E−08**	9
	rs1208	**3.80E−18**	**3.57E−25**	—	**2.40E−27**	**9.40E−20**	—	**9.00E−29**	**5.00E−24**	**4.10E−16**	**2.10E−28**	**1.50E−23**	9
	rs1799931	0.000212	**7.92E−11**	—	**3.30E−05**	**4.70E−06**	9.10E−04	**3.40E−10**	—	0.008694	**9.00E−10**	**8.70E−08**	6
*CYP2C19*	rs12248560	**1.10E−18**	**1.43E−21**	—	**6.20E−13**	**1.00E−12**	—	**1.90E−16**	**5.30E−10**	—	**1.80E−20**	**6.40E−22**	8
	rs4986893	—	—	—	—	—	—	—	—	—	—	—	0
	rs4244285	4.52E−02	5.29E−03	7.60E−03	—	—	—	—	1.59E−02	**2.00E−06**	**4.00E−05**	—	2
*CYP2C9*	rs1057910	—	—	—	—	2.26E−03	—	—	—	—	—	—	0
*CYP2C8*	rs7909236	—	**7.21E−05**	—	3.37E−02	1.24E−03	—	**6.40E−05**	**2.80E−05**	**2.30E−09**	—	**4.60E−07**	3
	rs17110453	**7.70E−08**	1.11E−04	1.74E−02	—	—	4.16E−04	**1.10E−12**	1.15E−02	**1.10E−06**	4.92E−04	**6.20E−13**	4
*CYP2E1*	rs2070676	**8.80E−13**	4.30E−02	—	—	—	—	**8.50E−30**	—	1.46E−02	—	**1.40E−22**	3
*GSTP1*	rs1695	**8.10E−07**	**9.74E−07**	—	—	1.06E−02	1.03E−02	**4.40E−13**	**1.00E−11**	**1.30E−21**	1.45E−02	**1.60E−06**	6
	rs1138272	—	—	—	**1.30E−08**	—	—	—	6.80E−05	—	—	—	1
*ANKK1*	rs1800497	—	2.04E−04	—	3.78E−02	—	—	—	—	—	6.56E−04	—	0
*DRD2*	rs6277	**8.80E−05**	**1.73E−36**	—	**2.70E−19**	**3.40E−20**	2.17E−02	—	**1.20E−14**	**3.00E−05**	**1.70E−44**	—	6
	rs1801028	4.39E−02	—	—	—	**2.70E−05**	—	2.17E−02	—	4.19E−02	—	4.22E−03	1
*SLCO1B1*	rs4149015	8.64E−03	—	—	1.81E−03	—	—	3.11E−02	4.65E−02	9.74E−04	—	3.30E−05	1
	rs2306283	—	**4.07E−14**	—	8.72E−11	8.03E−06	—	1.72E−02	**6.10E−13**	**1.70E−09**	**9.70E−17**	—	6
	rs4149056	—	1.26E−02	—	—	2.34E−02	—	3.00E−02	—	3.86E−02	**2.00E−06**	2.64E−03	1
*VDR*	rs731236	**1.70E−07**	**2.06E−23**	—	**1.00E−11**	**4.80E−14**	4.74E−02	**2.50E−10**	5.65E−05	—	**2.90E−21**	**6.40E−14**	7
	rs7975232	**4.90E−08**	**4.09E−08**	—	**3.40E−06**	**6.90E−06**	—	**2.20E−15**	—	3.29E−02	**5.20E−08**	**1.00E−08**	7
	rs1544410	**2.10E−10**	**1.04E−24**	—	**1.40E−23**	**2.70E−25**	—	**9.70E−11**	**1.70E−05**	1.73E−02	**1.50E−22**	**4.40E−13**	8
	rs2239185	1.95E−04	**2.35E−08**	—	**4.50E−06**	**1.00E−05**	—	**8.90E−11**	—	2.73E−02	**6.60E−08**	**8.50E−08**	6
	rs1540339	**7.90E−17**	**3.19E−16**	—	**6.50E−08**	**2.20E−14**	—	**3.50E−32**	**7.30E−10**	**9.40E−10**	**3.80E−14**	**8.00E−27**	9
	rs2239179	2.42E−02	**6.60E−11**	—	**1.20E−05**	**8.80E−10**	—	1.21E−02	—	—	5.18E−05	—	3
	rs3782905	2.21E−02	**2.84E−08**	—	1.45E−03	1.91E−03	—	5.37E−03	—	—	**4.90E−08**	2.65E−02	2
	rs4516035	—	**1.38E−17**	—	**6.40E−08**	**1.90E−05**	2.95E−03	—	**2.20E−08**	**3.10E−05**	**1.30E−22**	6.85E−03	6
	rs11568820	**1.90E−07**	**2.73E−05**	—	4.79E−02	—	—	**9.60E−20**	**3.50E−07**	**2.70E−12**	2.21E−04	**1.30E−35**	6
*CYP1A2*	rs762551	—	—	—	—	2.32E−02	—	0.000892	—	**1.30E−06**	—	—	1
*SULT1A1*	rs3760091	—	—	—	—	**2.90E−05**	—	—	—	**6.00E−08**	5.65E−04	—	2
*VKORC1*	rs7294	**2.80E−20**	**8.82E−13**	—	**2.50E−39**	**6.00E−43**	—	**3.80E−22**	**7.10E−13**	**7.80E−29**	**4.90E−15**	**1.40E−27**	9
	rs9934438	**2.40E−42**	**6.02E−29**	—	**1.10E−44**	**1.90E−49**	—	**3.70E−59**	**3.80E−24**	**1.20E−31**	**8.10E−28**	**6.40E−62**	9
*NQO1*	rs1800566	**1.10E−06**	**2.30E−08**	—	2.89E−02	—	—	**5.60E−09**	—	—	**4.10E−05**	**1.20E−08**	5
*CYP4F2*	rs2108622	**6.40E−05**	—	—	—	2.96E−03	—	**1.20E−05**	—	8.50E−05	—	**1.30E−10**	2
*CYP2A6*	rs8192726	—	—	1.67E−02	—	—	1.02E−02	—	—	—	—	—	0
	rs1801272	—	5.73E−04	—	—	—	—	—	3.68E−02	—	**5.00E−05**	—	1
	rs28399433	2.41E−02	—	**2.80E−05**	2.53E−04	2.02E−03	**2.30E−08**	6.02E−03	—	—	—	4.26E−04	2
	rs3211371	—	**9.79E−50**	—	—	—	—	—	—	—	**1.10E−48**	—	2
*PTGIS*	rs5629	—	—	—	—	—	—	5.39E−04	—	—	1.73E−02	—	0
*SLC19A1*	rs1051298	—	—	—	—	—	—	—	1.87E−02	8.11E−02	—	—	0
	rs1051296	—	—	—	—	—	—	—	1.08E−02	2.95E−02	—	—	0
	rs1051266	—	—	—	—	—	—	**3.90E−07**	3.29E−02	—	—	**2.30E−06**	2
	rs1131596	**4.10E−14**	**7.89E−22**	**5.70E−16**	**9.30E−14**	**5.30E−19**	**3.00E−14**	**4.10E−12**	**1.10E−09**	**3.20E−14**	5.80E−16	**4.70E−13**	11
*COMT*	rs4680	—	**1.25E−06**	—	**2.40E−08**	**2.30E−05**	—	—	1.62E−03	1.39E−03	**7.40E−07**	—	4
*CYP2D6*	rs59421388	1.28E−03	—	—	—	—	—	**8.20E−15**	—	—	—	**3.00E−10**	2
	rs28371725	—	1.43E−03	—	**7.70E−05**	2.42E−03	9.15E−03	—	—	2.47E−02	**1.80E−06**	1.83E−02	1
	rs61736512	1.28E−03	—	—			—	8.16E−15	—	—	—	**2.97E−10**	2
Different SNPs		3.20E + 01	35	3	35	35	3	40	24	24	35	43	

Note

ASW, African ancestry in southwestern USA; CEU, Utah residents with Northern and Western European ancestry; CHB, Han Chinese in Beijing, China; PJL, Punjabi in Lahore, Pakistan; GIH, Gujarati Indians in Houston, Texas, USA; JPT, Japanese in Tokyo, Japan; LWK, Luhya people in Webuye, Kenya; MXL, Mexican Ancestry in Los Angeles, Colombia; PEL, Peruvian in Lima, Peru; TSI, Toscans in Italy; YRI, Yoruba in Ibadan, Nigeria.

Bold italics indicates that after adjustment* p* < .05/(80*11) the locus has statistically significant.

### 3.3 Analyses of population genetic structures

The genetic differentiation degree of allele frequencies between Bai and other 11 populations was compared using Fst statistics. An Fst value of less than 0.15 indicates that there is no significant genetic difference between the two populations. And pairwise FST values between the Bai population and the other 11 HapMap populations ranged from 0.0157 to 0.2213 (Table 3 **)**. Comparing other populations, the lowest level of differentiation was observed between the Bai and CHB populations (FST = 0.0157), followed by the JPT (FST = 0.0203), whereas the greatest divergence was found in the YRI population (FST = 0.2213).

**Table 3:** Distribution of pairwise Fst distances among the Bai and all HapMap populations

Population	Bai	CHB	JPT	GIH	PJL	CEU	TSI	MXL	PEL	ASW	LWK	YRI
Bai	0.000											
CHB	0.016	0.000										
JPT	0.020	0.004	0.000									
GIH	0.129	0.128	0.116	0.000								
PJL	0.138	0.137	0.124	0.002	0.000							
CEU	0.146	0.149	0.140	0.038	0.030	0.000						
TSI	0.132	0.133	0.125	0.040	0.031	0.004	0.000					
MXL	0.109	0.108	0.107	0.049	0.040	0.030	0.026	0.000				
PEL	0.125	0.121	0.120	0.081	0.078	0.082	0.079	0.022	0.000			
ASW	0.174	0.178	0.164	0.088	0.085	0.115	0.112	0.098	0.110	0.000		
LWK	0.236	0.243	0.226	0.144	0.142	0.177	0.174	0.167	0.176	0.013	0.000	
YRI	0.221	0.227	0.209	0.139	0.137	0.179	0.175	0.165	0.170	0.009	0.008	0.000

Note

ASW, African ancestry in southwestern USA; CEU, Utah residents with Northern and Western European ancestry; CHB, Han Chinese in Beijing, China; PJL, Punjabi in Lahore, Pakistan; GIH, Gujarati Indians in Houston, Texas, USA; JPT, Japanese in Tokyo, Japan; LWK, Luhya people in Webuye, Kenya; MXL, Mexican Ancestry in Los Angeles, Colombia; PEL, Peruvian in Lima, Peru; TSI, Toscans in Italy; YRI, Yoruba in Ibadan, Nigeria.

**Figure 1:**
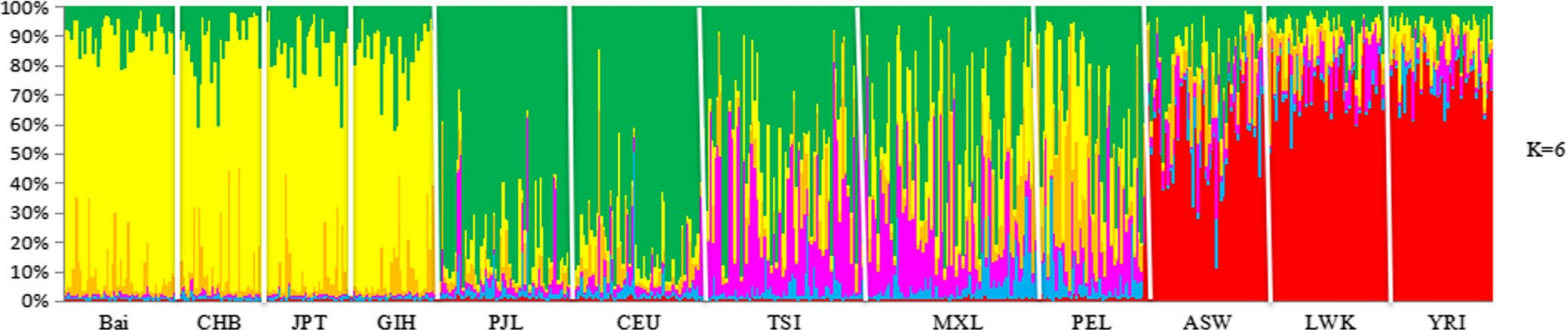
Results of structure clustering analysis (*K* = 6) for Bai and HapMap populations

The Bayesian‐based structure analysis of the genetic relationship among 12 populations was shown in Figure 1, most suitable *K* was observed at *K* = 6, where the each individual was represented by a vertical column partitioned into different color segments. The results revealed that the Bai population was most similar to the CHB and JPT populations, which was consistent with the results in Table 3.

## 4 DISCUSSION

Today, the rapidly advancing pharmacogenetics is increasingly focused on the interethnic or interracial differences in drug metabolism to identify the genetic backgrounds of these variations. For the first time, our study genotyped the variants related to drug reactions in the Bai ethnic group, and compared the genotype frequencies with the other 11 HapMap populations. Our results suggested that the expression of many VIP variants were significantly different between the Bai population and other populations. Among these variants, rs20417, rs4148323, and rs1131596 were significantly differentially expressed in the Bai population, compared to the 11 populations. We also found that the genetic backgrounds of the Bai and CHB populations were similar, but significantly different from the YRI populations.

Rs20417 is a significant variant of the prostaglandin‐endoperoxide synthase 2 (*PTGS2*) gene (Hung et al., 2017). *PTGS2* gene also known as cyclooxygenase 2 (*COX‐2*), located on chromosome 1, is the key enzyme in prostaglandin biosynthesis that can converts arachidonate to prostaglandin H2 (*PGH2*) (Lucido, Orlando, Vecchio, & Malkowski, 2016). It has been widely reported that the polymorphism of rs20417 is associated with many diseases, such as myocardial infarction or stroke (Lemaitre et al., 2009). Previous pharmacogenomics studies have revealed that *PTGS2* was the targets of nonsteroidal anti‐inflammatory drugs (*NSAIDs*) including aspirin and ibuprofen (Orlando & Malkowski, 2016). Studies by Yun‐Sil Lee DDS et al. (Y. S. Lee, Kim, Wu, Wang, & Dionne, 2006) showed that CC genotype patients tend to increase pain relief when treated with rofecoxib compared to genotype GG + CG, but reduce pain relief with ibuprofen. Lee C R et al. (C. R. Lee et al., 2008) also reported that patients with a CC genotype may have an increased risk of coronary artery disease when treated with aspirin compared to patients with a GG or CG genotype. Rozenn et al. (Lemaitre et al., 2009) studies have shown that that variation in *TBXAS1* and *PTGIS* may influence Myocardial Infarction (MI) risk and carriers of rs20417 C allele might derive greater benefits from aspirin use in primary prevention in comparison with non‐carriers. In our study, the C allele frequency of the Bai nationality was as high as 80%, indicating that the Bai population increased the risk of coronary artery disease when treated with aspirin. At the same time, when using aspirin to prevent MI risk, the Bai population can get more benefits.

Rs4148323 is an intron variant of the *UGT1A1* gene on human chromosome 2q37, which encodes a UDP‐glucuronosyltransferase, an enzyme of the glucuronidation pathway (Sugatani et al., 2001). It plays an important role in catalyzing the formation of bound bilirubin by unbound bilirubin (Liu, Lu, et al., 2017). Clinical studies have found that cancer patients carrying the GG genotype may reduce the risk of thrombocytopenia (Han, Lim, Park, Lee, & Lee, 2009) or diarrhea (Takekuma et al., 2006) when treated with irinotecan‐based regimens and may also increase tumor response, progression‐free survival Period or overall survival compared with patients with AA or AG genotype. In our study, we found that the G allele frequency of rs 4,148,323 in the Bai population was very high, indicating that the cancer patients in the Bai population can reduce the risk of thrombocytopenia or diarrhea. Other studies have demonstrated that patients with angina or heart failure carrying the G allele are more likely to increase glucuronidation of carvedilol than carriers of the A allele (Boyd et al., 2006). One study reported that patients with the G allele had a reduced risk of developing hyperbilirubinemia during treatment with indinavir, compared to HIV patients with the A allele (Bohanec Grabar et al., 2012). These findings pointed that rs4148323 polymorphism may be a useful pharmacogenomics point for providing rational and effectively tailored therapy for the Bai ethnic group.

The rs1131596 variant is located in the Solute Carrier Family 19 Member 1 (*SLC19A1*) gene, which encodes a folate transporter and is involved in the regulation of intracellular folate concentrations (Whetstine, Flatley, & Matherly, 2002). In our study, we found that the genotype frequency of rs1131596 was significantly different between the Bai and the other 11 races. One study reported that a linkage group (LD) rs1051266/rs11315962, which may influence the *SLC19A1* function, such as changing the SLC19A1 splicing (Bohanec Grabar et al., 2012). Clinical evidence proposed that variants of rs1131596 and rs1051266 have protective effects against the risk of discontinuation of methotrexate toxicity (MTX) treatment due to toxicity and infection (Chatzikyriakidou et al., 2007). The allele G of rs1131596 can reduce the express‐

ion of *SLC19A1* compared to allele A, but the allele G was not associated with response to methotrexate in people with arthritis, rheumatoid and in children with progenitor cell lymphoblastic leukemia‐lymphoma (Liu, Gao, et al., 2017). However, the pharmacoge‐ nomics information of the rs3807375 variant requires more in‐depth investigation.

Our results supplemented the pharmacogenomic information of the Bai population and shed light on the differences in selected genetic polymorphisms between the Bai population and 11 other populations around the world. In addition, these results provided a solid foundation for the Bai population to use drugs more rationally and safely. But our sample of the Bai population was relatively small and the results must be further validated in a larger sample set.

## 5 CONCLUSIONS

We identified the characteristics of 81 VIP variants of Bai population from southwestern China, and found that the genetic background of Bai population in Yunnan was closest to CHD population. This information helps Bai population to develop appropriate personalized treatment strategies, including appropriate drugs and the right dose.

## 6 THE INFLUENCE OF OUR RESULTS IN CLINICAL APPLICATION

Different populations may have different genotypes due to differences in ancestry, geographical location, lifestyle, etc., and different genotypes have certain differences in response to corresponding drugs. Our study found that the genotypes of some VIP sites of Bai population were different from those of 11 global representative groups, so this study is helpful for the individualized treatment of Bai population in clinical practice.

## 7 ETHICS APPROVAL AND CONSENT TO PARTICIPATE

All volunteers were informed the procedures and purpose of the study, both orally and in writing. They also agreed to provide blood samples and signed informed consent forms. The clinical protocol was approved by the Ethics Committee of Yunnan First People's Hospital and was performed in accordance with the Declaration of Helsinki.

## 8 CONSENT FOR PUBLICATION

Not applicable.

## CONFLICT OF INTERESTS

The authors declare that they have no competing interests.

## AUTHORS’ CONTRIBUTIONS

Wanlu Chen and Heng Ding: conceived and designed the experiments. Yujing Cheng and Qi Li: performed the experiments. Run Dai and Xin Yang: analyzed the data. Chan Zhang: contributed reagents/materials/analysis tools.

9

## Data Availability

The datasets used or analyzed during the current study are available from the corresponding author on reasonable request.
