# Supplementary material for: Genetic polymorphisms analysis of pharmacogenomic VIP variants in Bai ethnic group from China
Source: Mol Genet Genomic Med. 2019 Jul 30;7(9):e884. doi: 10.1002/mgg3.884 (PMC6732286; doi:10.1002/mgg3.884)
Supplement: Supplementary file 1 [file MGG3-7-e884-s001.docx]

**Table S1**. The genotype count of 81 loci in HapMap 11 populations

| SNP-ID | CHB | | | JPT | | | GIH | | | PJL | | | CEU | | | TSI | | | MXL | | | PEL | | | ASW | | | LWK | | | YRI | | |
| --- | --- | --- | --- | --- | --- | --- | --- | --- | --- | --- | --- | --- | --- | --- | --- | --- | --- | --- | --- | --- | --- | --- | --- | --- | --- | --- | --- | --- | --- | --- | --- | --- | --- |
|  | AA | AB | BB | AA | AB | BB | AA | AB | BB | AA | AB | BB | AA | AB | BB | AA | AB | BB | AA | AB | BB | AA | AB | BB | AA | AB | BB | AA | AB | BB | AA | AB | BB |
| rs1801131 | 4 | 38 | 61 | 3 | 31 | 70 | 18 | 50 | 35 | 16 | 46 | 31 | 12 | 41 | 46 | 12 | 43 | 52 | 1 | 19 | 44 | 1 | 12 | 72 | 2 | 21 | 38 | 5 | 27 | 67 | 0 | 26 | 82 |
| rs1801133 | 23 | 50 | 30 | 15 | 49 | 40 | 2 | 27 | 74 | 0 | 25 | 68 | 10 | 39 | 50 | 26 | 48 | 33 | 13 | 34 | 17 | 16 | 42 | 27 | 4 | 9 | 48 | 0 | 14 | 85 | 0 | 23 | 85 |
| rs890293 | 1 | 7 | 95 | 0 | 5 | 99 | 0 | 11 | 92 | 0 | 12 | 81 | 0 | 10 | 89 | 0 | 11 | 96 | 0 | 4 | 60 | 0 | 1 | 84 | 3 | 14 | 44 | 1 | 25 | 73 | 4 | 25 | 79 |
| rs3918290 | 0 | 0 | 103 | 0 | 0 | 104 | 0 | 3 | 100 | 0 | 1 | 92 | 0 | 1 | 98 | 0 | 1 | 106 | 0 | 0 | 64 | 0 | 1 | 84 | 0 | 1 | 60 | 0 | 0 | 99 | 0 | 0 | 108 |
| rs1801159 | 6 | 43 | 54 | 9 | 36 | 59 | 0 | 21 | 82 | 0 | 16 | 77 | 1 | 29 | 69 | 10 | 33 | 64 | 2 | 25 | 37 | 20 | 40 | 25 | 1 | 16 | 44 | 8 | 44 | 47 | 2 | 28 | 78 |
| rs1801265 | 0 | 14 | 89 | 1 | 10 | 93 | 7 | 53 | 43 | 8 | 39 | 46 | 3 | 25 | 71 | 7 | 33 | 67 | 6 | 19 | 39 | 3 | 22 | 60 | 13 | 33 | 15 | 27 | 45 | 27 | 18 | 58 | 32 |
| rs6025 | 0 | 0 | 103 | 0 | 0 | 104 | 0 | 1 | 102 | 0 | 2 | 91 | 1 | 3 | 95 | 0 | 1 | 106 | 0 | 1 | 63 | 0 | 2 | 83 | 0 | 0 | 61 | 0 | 0 | 99 | 0 | 0 | 108 |
| rs5275 | 0 | 36 | 67 | 4 | 42 | 58 | 13 | 52 | 38 | 23 | 42 | 28 | 11 | 53 | 35 | 11 | 43 | 53 | 9 | 28 | 27 | 10 | 47 | 28 | 18 | 34 | 9 | 40 | 44 | 15 | 46 | 52 | 10 |
| rs20417 | 0 | 11 | 92 | 0 | 9 | 95 | 1 | 32 | 70 | 6 | 31 | 56 | 3 | 28 | 68 | 4 | 32 | 71 | 1 | 25 | 38 | 5 | 25 | 55 | 5 | 28 | 28 | 9 | 38 | 52 | 16 | 49 | 43 |
| rs689466 | 22 | 53 | 28 | 26 | 40 | 38 | 1 | 24 | 78 | 4 | 17 | 72 | 7 | 24 | 68 | 2 | 38 | 67 | 4 | 25 | 35 | 3 | 50 | 32 | 1 | 14 | 46 | 0 | 6 | 93 | 0 | 16 | 92 |
| rs4124874 | 4 | 48 | 51 | 11 | 43 | 50 | 35 | 55 | 13 | 38 | 47 | 8 | 19 | 48 | 32 | 20 | 55 | 32 | 18 | 30 | 16 | 32 | 39 | 14 | 35 | 25 | 1 | 78 | 21 | 0 | 89 | 18 | 1 |
| rs10929302 | 0 | 23 | 80 | 6 | 25 | 73 | 24 | 42 | 37 | 15 | 49 | 29 | 8 | 45 | 46 | 7 | 37 | 63 | 6 | 32 | 26 | 16 | 45 | 24 | 1 | 38 | 22 | 10 | 51 | 38 | 15 | 48 | 45 |
| rs4148323 | 2 | 43 | 58 | 2 | 23 | 79 | 0 | 4 | 99 | 0 | 0 | 93 | 0 | 0 | 99 | 0 | 0 | 107 | 0 | 3 | 61 | 0 | 0 | 85 | 0 | 1 | 60 | 0 | 0 | 99 | 0 | 0 | 108 |
| rs1805124 | 0 | 26 | 77 | 1 | 26 | 77 | 2 | 38 | 63 | 5 | 39 | 49 | 2 | 32 | 65 | 5 | 40 | 62 | 2 | 17 | 45 | 6 | 26 | 53 | 5 | 22 | 34 | 9 | 42 | 48 | 9 | 53 | 46 |
| rs6791924 | 0 | 0 | 103 | 0 | 0 | 104 | 0 | 0 | 103 | 0 | 0 | 93 | 0 | 0 | 99 | 0 | 0 | 107 | 0 | 3 | 61 | 0 | 1 | 84 | 0 | 7 | 54 | 3 | 26 | 70 | 1 | 17 | 90 |
| rs3814055 | 9 | 38 | 56 | 9 | 35 | 60 | 18 | 50 | 35 | 7 | 44 | 42 | 12 | 43 | 44 | 14 | 49 | 44 | 5 | 33 | 26 | 14 | 38 | 33 | 4 | 28 | 29 | 11 | 38 | 50 | 11 | 37 | 60 |
| rs2046934 | 6 | 30 | 67 | 1 | 29 | 74 | 1 | 19 | 83 | 2 | 21 | 70 | 3 | 34 | 62 | 2 | 22 | 83 | 1 | 12 | 51 | 0 | 12 | 73 | 1 | 15 | 45 | 1 | 18 | 80 | 3 | 29 | 76 |
| rs1065776 | 0 | 7 | 96 | 0 | 15 | 89 | 0 | 21 | 82 | 3 | 22 | 68 | 1 | 9 | 89 | 1 | 5 | 101 | 0 | 6 | 58 | 0 | 11 | 74 | 2 | 21 | 38 | 4 | 34 | 61 | 7 | 34 | 67 |
| rs701265 | 9 | 41 | 53 | 9 | 39 | 56 | 3 | 37 | 63 | 6 | 33 | 54 | 3 | 27 | 69 | 4 | 27 | 76 | 3 | 20 | 41 | 9 | 35 | 41 | 28 | 26 | 7 | 63 | 34 | 2 | 71 | 33 | 4 |
| rs975833 | 2 | 39 | 62 | 3 | 35 | 66 | 24 | 57 | 22 | 31 | 47 | 15 | 57 | 36 | 6 | 55 | 46 | 6 | 48 | 15 | 1 | 67 | 18 | 0 | 33 | 25 | 3 | 62 | 36 | 1 | 51 | 49 | 8 |
| rs2066702 | 0 | 0 | 103 | 0 | 0 | 104 | 0 | 0 | 103 | 0 | 0 | 93 | 0 | 0 | 99 | 0 | 0 | 107 | 0 | 4 | 60 | 0 | 2 | 83 | 3 | 19 | 39 | 0 | 28 | 71 | 6 | 49 | 53 |
| rs698 | 0 | 10 | 93 | 1 | 13 | 90 | 10 | 38 | 55 | 10 | 41 | 42 | 20 | 54 | 25 | 11 | 44 | 52 | 4 | 28 | 32 | 2 | 26 | 57 | 2 | 13 | 46 | 1 | 26 | 72 | 0 | 15 | 93 |
| rs17244841 | 0 | 0 | 103 | 0 | 4 | 100 | 0 | 0 | 103 | 0 | 0 | 93 | 0 | 3 | 96 | 1 | 5 | 101 | 0 | 5 | 59 | 0 | 1 | 84 | 0 | 12 | 49 | 1 | 15 | 83 | 1 | 18 | 89 |
| rs3846662 | 29 | 48 | 26 | 26 | 58 | 20 | 44 | 48 | 11 | 31 | 45 | 17 | 18 | 48 | 33 | 19 | 54 | 34 | 13 | 30 | 21 | 27 | 41 | 17 | 47 | 12 | 2 | 93 | 6 | 0 | 99 | 9 | 0 |
| rs1042713 | 17 | 59 | 27 | 33 | 50 | 21 | 35 | 47 | 21 | 30 | 45 | 18 | 40 | 49 | 10 | 45 | 45 | 17 | 16 | 35 | 13 | 31 | 34 | 20 | 13 | 29 | 19 | 27 | 47 | 25 | 27 | 47 | 34 |
| rs1042714 | 0 | 22 | 81 | 1 | 10 | 93 | 3 | 41 | 59 | 8 | 27 | 58 | 23 | 46 | 30 | 14 | 56 | 37 | 2 | 14 | 48 | 3 | 17 | 65 | 1 | 13 | 47 | 1 | 40 | 58 | 2 | 22 | 84 |
| rs1142345 | 0 | 1 | 102 | 0 | 4 | 100 | 0 | 5 | 98 | 0 | 2 | 91 | 0 | 6 | 93 | 0 | 3 | 104 | 0 | 6 | 58 | 0 | 11 | 74 | 0 | 12 | 49 | 3 | 17 | 79 | 1 | 11 | 96 |
| rs2066853 | 15 | 48 | 40 | 22 | 52 | 30 | 3 | 18 | 82 | 2 | 23 | 68 | 0 | 18 | 81 | 0 | 21 | 86 | 0 | 17 | 47 | 6 | 26 | 53 | 5 | 32 | 24 | 23 | 51 | 25 | 23 | 52 | 33 |
| rs1045642 | 18 | 42 | 43 | 21 | 58 | 25 | 34 | 49 | 20 | 24 | 47 | 22 | 31 | 50 | 18 | 25 | 50 | 32 | 13 | 35 | 16 | 10 | 44 | 31 | 3 | 17 | 41 | 1 | 26 | 72 | 3 | 21 | 84 |
| rs1128503 | 11 | 40 | 52 | 18 | 47 | 39 | 17 | 55 | 31 | 22 | 43 | 28 | 29 | 55 | 15 | 38 | 48 | 21 | 17 | 34 | 13 | 35 | 44 | 6 | 41 | 19 | 1 | 78 | 20 | 1 | 78 | 29 | 1 |
| rs2740574 | 0 | 0 | 103 | 0 | 0 | 104 | 0 | 16 | 87 | 0 | 5 | 88 | 0 | 3 | 96 | 0 | 6 | 101 | 0 | 9 | 55 | 0 | 6 | 79 | 26 | 30 | 5 | 72 | 21 | 6 | 63 | 39 | 6 |
| rs3807375 | 7 | 37 | 59 | 2 | 37 | 65 | 38 | 50 | 15 | 35 | 44 | 14 | 41 | 47 | 11 | 43 | 55 | 9 | 13 | 29 | 22 | 7 | 34 | 44 | 3 | 27 | 31 | 8 | 24 | 67 | 6 | 37 | 65 |
| rs4646244 | 4 | 32 | 67 | 10 | 35 | 59 | 13 | 51 | 39 | 11 | 41 | 41 | 13 | 37 | 49 | 8 | 44 | 55 | 0 | 16 | 48 | 0 | 15 | 70 | 5 | 22 | 34 | 5 | 38 | 56 | 4 | 35 | 69 |
| rs4271002 | 1 | 37 | 65 | 6 | 30 | 68 | 2 | 24 | 77 | 2 | 22 | 69 | 0 | 14 | 85 | 4 | 28 | 75 | 2 | 20 | 42 | 8 | 23 | 54 | 1 | 12 | 48 | 0 | 17 | 82 | 1 | 16 | 91 |
| rs1801280 | 0 | 6 | 97 | 0 | 4 | 100 | 15 | 41 | 47 | 16 | 46 | 31 | 19 | 46 | 34 | 21 | 50 | 36 | 11 | 27 | 26 | 4 | 39 | 42 | 7 | 24 | 30 | 11 | 49 | 39 | 8 | 36 | 64 |
| rs1799929 | 0 | 6 | 97 | 0 | 4 | 100 | 10 | 42 | 51 | 15 | 43 | 35 | 17 | 48 | 34 | 21 | 50 | 36 | 11 | 26 | 27 | 3 | 39 | 43 | 5 | 22 | 34 | 10 | 45 | 44 | 6 | 25 | 77 |
| rs1208 | 0 | 6 | 97 | 0 | 4 | 100 | 15 | 41 | 47 | 17 | 46 | 30 | 16 | 47 | 36 | 21 | 51 | 35 | 14 | 29 | 21 | 4 | 39 | 42 | 9 | 26 | 26 | 23 | 45 | 31 | 15 | 50 | 43 |
| rs1799931 | 0 | 34 | 69 | 3 | 15 | 86 | 0 | 11 | 92 | 0 | 11 | 82 | 0 | 1 | 98 | 0 | 4 | 103 | 0 | 18 | 46 | 7 | 16 | 62 | 0 | 6 | 55 | 0 | 2 | 97 | 0 | 8 | 100 |
| rs12248560 | 0 | 5 | 98 | 0 | 1 | 103 | 2 | 24 | 77 | 3 | 21 | 69 | 3 | 38 | 58 | 6 | 36 | 65 | 2 | 11 | 51 | 0 | 7 | 78 | 1 | 22 | 38 | 3 | 29 | 67 | 8 | 37 | 63 |
| rs4986893 | 0 | 9 | 94 | 1 | 13 | 90 | 0 | 1 | 102 | 0 | 3 | 90 | 0 | 0 | 99 | 0 | 0 | 107 | 0 | 0 | 64 | 0 | 0 | 85 | 0 | 0 | 61 | 0 | 2 | 97 | 0 | 0 | 108 |
| rs4244285 | 7 | 55 | 41 | 11 | 45 | 48 | 15 | 38 | 50 | 15 | 33 | 45 | 2 | 22 | 75 | 1 | 18 | 88 | 1 | 14 | 49 | 0 | 10 | 75 | 1 | 15 | 45 | 5 | 32 | 62 | 5 | 26 | 77 |
| rs1057910 | 0 | 8 | 95 | 0 | 4 | 100 | 1 | 25 | 77 | 1 | 17 | 75 | 0 | 13 | 86 | 1 | 16 | 90 | 0 | 3 | 61 | 0 | 2 | 83 | 0 | 2 | 59 | 0 | 0 | 99 | 0 | 0 | 108 |
| rs7909236 | 1 | 19 | 83 | 0 | 15 | 89 | 5 | 39 | 59 | 6 | 25 | 62 | 5 | 42 | 52 | 3 | 32 | 72 | 5 | 28 | 31 | 11 | 39 | 35 | 0 | 10 | 51 | 0 | 3 | 96 | 0 | 0 | 108 |
| rs17110453 | 11 | 48 | 44 | 14 | 52 | 38 | 13 | 39 | 51 | 8 | 33 | 52 | 0 | 17 | 82 | 2 | 18 | 87 | 1 | 12 | 51 | 0 | 8 | 77 | 0 | 1 | 60 | 0 | 0 | 99 | 0 | 1 | 107 |
| rs2070676 | 2 | 36 | 65 | 3 | 36 | 65 | 2 | 26 | 75 | 2 | 26 | 65 | 0 | 28 | 71 | 0 | 36 | 71 | 4 | 12 | 48 | 3 | 15 | 67 | 21 | 29 | 11 | 56 | 39 | 4 | 45 | 52 | 11 |
| rs1695 | 2 | 34 | 67 | 2 | 17 | 85 | 10 | 44 | 49 | 13 | 28 | 52 | 12 | 54 | 33 | 8 | 47 | 52 | 22 | 28 | 14 | 35 | 44 | 6 | 13 | 30 | 18 | 22 | 57 | 20 | 16 | 54 | 38 |
| rs1138272 | 0 | 0 | 103 | 0 | 0 | 104 | 0 | 17 | 86 | 1 | 15 | 77 | 0 | 19 | 80 | 0 | 11 | 96 | 1 | 5 | 58 | 0 | 1 | 84 | 0 | 3 | 58 | 0 | 3 | 96 | 0 | 0 | 108 |
| rs1800497 | 24 | 42 | 37 | 15 | 51 | 38 | 9 | 41 | 53 | 10 | 31 | 52 | 5 | 30 | 64 | 7 | 33 | 67 | 10 | 36 | 18 | 20 | 34 | 31 | 12 | 29 | 20 | 12 | 51 | 36 | 17 | 51 | 40 |
| rs6277 | 0 | 8 | 95 | 0 | 16 | 88 | 13 | 43 | 47 | 13 | 37 | 43 | 21 | 57 | 21 | 35 | 58 | 14 | 9 | 22 | 33 | 2 | 20 | 63 | 2 | 14 | 45 | 0 | 3 | 96 | 0 | 6 | 102 |
| rs1801028 | 1 | 8 | 94 | 0 | 5 | 99 | 1 | 30 | 72 | 0 | 7 | 86 | 1 | 5 | 93 | 0 | 4 | 103 | 0 | 3 | 61 | 0 | 1 | 84 | 0 | 0 | 61 | 0 | 1 | 98 | 0 | 0 | 108 |
| rs4149015 | 1 | 23 | 79 | 1 | 22 | 81 | 0 | 12 | 91 | 1 | 2 | 90 | 0 | 8 | 91 | 0 | 17 | 90 | 0 | 3 | 61 | 0 | 1 | 84 | 0 | 1 | 60 | 0 | 6 | 93 | 0 | 0 | 108 |
| rs2306283 | 6 | 34 | 63 | 12 | 47 | 45 | 22 | 48 | 33 | 29 | 44 | 20 | 36 | 46 | 17 | 34 | 62 | 11 | 24 | 32 | 8 | 22 | 46 | 17 | 4 | 23 | 34 | 5 | 21 | 73 | 3 | 34 | 71 |
| rs4149056 | 2 | 24 | 77 | 3 | 19 | 82 | 0 | 4 | 99 | 1 | 5 | 87 | 1 | 27 | 71 | 4 | 38 | 65 | 0 | 10 | 54 | 2 | 20 | 63 | 1 | 6 | 54 | 0 | 4 | 95 | 0 | 2 | 106 |
| rs731236 | 0 | 8 | 95 | 0 | 24 | 80 | 9 | 47 | 47 | 11 | 35 | 47 | 25 | 43 | 31 | 19 | 51 | 37 | 2 | 22 | 40 | 2 | 16 | 67 | 2 | 26 | 33 | 6 | 41 | 52 | 9 | 49 | 50 |
| rs7975232 | 10 | 38 | 55 | 11 | 48 | 45 | 22 | 62 | 19 | 27 | 49 | 17 | 40 | 38 | 21 | 39 | 48 | 20 | 9 | 34 | 21 | 6 | 25 | 54 | 21 | 35 | 5 | 51 | 40 | 8 | 42 | 46 | 20 |
| rs1544410 | 0 | 9 | 94 | 0 | 25 | 79 | 15 | 58 | 30 | 18 | 46 | 29 | 25 | 43 | 31 | 19 | 51 | 37 | 2 | 21 | 41 | 2 | 17 | 66 | 2 | 29 | 30 | 4 | 42 | 53 | 11 | 42 | 55 |
| rs2239185 | 10 | 38 | 55 | 10 | 48 | 46 | 21 | 63 | 19 | 27 | 49 | 17 | 41 | 37 | 21 | 39 | 48 | 20 | 10 | 33 | 21 | 6 | 25 | 54 | 18 | 31 | 12 | 37 | 52 | 10 | 37 | 52 | 19 |
| rs1540339 | 8 | 39 | 56 | 8 | 36 | 60 | 47 | 40 | 16 | 32 | 38 | 23 | 48 | 39 | 12 | 46 | 46 | 15 | 27 | 25 | 12 | 28 | 45 | 12 | 33 | 26 | 2 | 74 | 24 | 1 | 70 | 33 | 5 |
| rs2239179 | 4 | 38 | 61 | 6 | 30 | 68 | 27 | 50 | 26 | 21 | 36 | 36 | 34 | 32 | 33 | 17 | 54 | 36 | 6 | 26 | 32 | 3 | 23 | 59 | 3 | 35 | 23 | 12 | 46 | 41 | 10 | 43 | 55 |
| rs3782905 | 2 | 26 | 75 | 1 | 17 | 86 | 8 | 36 | 59 | 10 | 29 | 54 | 16 | 39 | 44 | 12 | 48 | 47 | 3 | 21 | 40 | 2 | 15 | 68 | 2 | 23 | 36 | 4 | 37 | 58 | 6 | 35 | 67 |
| rs4516035 | 0 | 5 | 98 | 0 | 1 | 103 | 3 | 33 | 67 | 4 | 35 | 54 | 15 | 44 | 40 | 17 | 55 | 35 | 5 | 24 | 35 | 8 | 18 | 59 | 1 | 12 | 48 | 0 | 9 | 90 | 0 | 2 | 106 |
| rs11568820 | 20 | 46 | 37 | 24 | 47 | 33 | 16 | 48 | 39 | 13 | 33 | 47 | 5 | 34 | 60 | 7 | 39 | 61 | 2 | 15 | 47 | 2 | 13 | 70 | 29 | 30 | 2 | 72 | 26 | 1 | 103 | 5 | 0 |
| rs762551 | 15 | 45 | 43 | 17 | 49 | 38 | 24 | 50 | 29 | 19 | 47 | 27 | 8 | 38 | 53 | 16 | 49 | 42 | 8 | 18 | 38 | 2 | 19 | 64 | 7 | 30 | 24 | 26 | 51 | 22 | 23 | 53 | 32 |
| rs3760091 | 17 | 47 | 39 | 13 | 55 | 36 | 3 | 38 | 62 | 14 | 42 | 37 | 15 | 50 | 34 | 28 | 49 | 30 | 13 | 35 | 16 | 4 | 21 | 60 | 5 | 36 | 20 | 8 | 52 | 39 | 7 | 62 | 39 |
| rs7294 | 0 | 9 | 94 | 0 | 20 | 84 | 39 | 59 | 5 | 48 | 36 | 9 | 9 | 44 | 46 | 11 | 50 | 46 | 8 | 29 | 27 | 28 | 40 | 17 | 14 | 30 | 17 | 16 | 53 | 30 | 30 | 51 | 27 |
| rs9934438 | 0 | 9 | 94 | 0 | 20 | 84 | 68 | 34 | 1 | 64 | 23 | 6 | 36 | 41 | 22 | 30 | 52 | 25 | 17 | 34 | 13 | 32 | 40 | 13 | 46 | 12 | 3 | 92 | 7 | 0 | 102 | 6 | 0 |
| rs1800566 | 20 | 63 | 20 | 8 | 56 | 40 | 20 | 38 | 45 | 8 | 42 | 43 | 3 | 30 | 66 | 8 | 37 | 62 | 10 | 28 | 26 | 15 | 41 | 29 | 2 | 17 | 42 | 2 | 30 | 67 | 4 | 32 | 72 |
| rs2108622 | 4 | 37 | 62 | 10 | 28 | 66 | 21 | 48 | 34 | 15 | 42 | 36 | 8 | 33 | 58 | 17 | 38 | 52 | 2 | 28 | 34 | 0 | 20 | 65 | 0 | 11 | 50 | 1 | 20 | 78 | 1 | 10 | 97 |
| rs8192726 | 7 | 25 | 71 | 8 | 24 | 72 | 2 | 24 | 77 | 5 | 21 | 67 | 0 | 10 | 89 | 0 | 14 | 93 | 0 | 6 | 58 | 0 | 8 | 77 | 1 | 10 | 50 | 1 | 16 | 82 | 3 | 16 | 89 |
| rs1801272 | 0 | 0 | 103 | 0 | 0 | 104 | 0 | 2 | 101 | 0 | 0 | 93 | 0 | 7 | 92 | 0 | 10 | 97 | 0 | 2 | 62 | 0 | 2 | 83 | 0 | 1 | 60 | 0 | 0 | 99 | 0 | 0 | 108 |
| rs28399433 | 10 | 35 | 58 | 17 | 24 | 63 | 6 | 27 | 70 | 7 | 22 | 64 | 0 | 10 | 89 | 0 | 14 | 93 | 0 | 13 | 51 | 0 | 16 | 69 | 1 | 11 | 49 | 1 | 16 | 82 | 3 | 16 | 89 |
| rs3211371 | 0 | 0 | 103 | 0 | 2 | 102 | 0 | 20 | 83 | 0 | 18 | 75 | 1 | 17 | 81 | 1 | 21 | 85 | 0 | 11 | 53 | 0 | 5 | 80 | 0 | 4 | 57 | 0 | 0 | 99 | 0 | 2 | 106 |
| rs5629 | 2 | 44 | 57 | 3 | 39 | 62 | 3 | 42 | 58 | 7 | 39 | 47 | 6 | 34 | 59 | 12 | 47 | 48 | 6 | 28 | 30 | 3 | 36 | 46 | 2 | 15 | 44 | 0 | 18 | 81 | 3 | 31 | 74 |
| rs1051298 | 27 | 49 | 27 | 34 | 49 | 21 | 37 | 48 | 18 | 24 | 48 | 21 | 14 | 52 | 33 | 20 | 54 | 33 | 11 | 24 | 29 | 18 | 33 | 34 | 19 | 29 | 13 | 31 | 45 | 23 | 34 | 54 | 20 |
| rs1051296 | 29 | 47 | 27 | 34 | 49 | 21 | 28 | 53 | 22 | 23 | 45 | 25 | 14 | 52 | 33 | 20 | 53 | 34 | 11 | 24 | 29 | 18 | 32 | 35 | 14 | 29 | 18 | 31 | 42 | 26 | 24 | 57 | 27 |
| rs1051266 | 24 | 50 | 29 | 32 | 49 | 23 | 15 | 51 | 37 | 19 | 39 | 35 | 14 | 57 | 28 | 23 | 50 | 34 | 12 | 21 | 31 | 14 | 35 | 36 | 19 | 31 | 11 | 46 | 45 | 8 | 49 | 47 | 12 |
| rs1131596 | 29 | 50 | 24 | 23 | 49 | 32 | 37 | 51 | 15 | 35 | 39 | 19 | 28 | 57 | 14 | 34 | 50 | 23 | 31 | 21 | 12 | 36 | 35 | 14 | 7 | 32 | 22 | 5 | 37 | 57 | 8 | 44 | 56 |
| rs4680 | 12 | 41 | 50 | 7 | 45 | 52 | 23 | 44 | 36 | 31 | 32 | 30 | 23 | 46 | 30 | 21 | 55 | 31 | 9 | 33 | 22 | 7 | 46 | 32 | 7 | 19 | 35 | 7 | 43 | 49 | 11 | 44 | 53 |
| rs59421388 | 0 | 0 | 103 | 0 | 0 | 104 | 0 | 0 | 103 | 0 | 0 | 93 | 0 | 0 | 99 | 0 | 0 | 107 | 0 | 0 | 64 | 0 | 0 | 85 | 1 | 3 | 57 | 5 | 24 | 70 | 1 | 21 | 86 |
| rs28371725 | 0 | 7 | 96 | 0 | 1 | 103 | 5 | 20 | 78 | 0 | 21 | 72 | 2 | 20 | 77 | 1 | 29 | 77 | 0 | 2 | 62 | 0 | 1 | 84 | 0 | 2 | 59 | 0 | 6 | 93 | 0 | 2 | 106 |
| rs61736512 | 0 | 0 | 103 | 0 | 0 | 104 | 0 | 0 | 103 | 0 | 0 | 93 | 0 | 0 | 99 | 0 | 0 | 107 | 0 | 0 | 64 | 0 | 0 | 85 | 1 | 3 | 57 | 5 | 24 | 70 | 2 | 20 | 86 |

ASW (n = 61); CEU (n = 99); CHB (n = 103); PJL (n = 93); GIH (n = 103); JPT (n = 104); LWK (n = 99); MXL (n = 64); PEL (n=85); TSI (n = 107); YRI (n = 108); ASW: African ancestry in southwestern USA; CEU: Utah residents with Northern and Western European ancestry; CHB: Han Chinese in Beijing, China; PJL: Punjabi in Lahore, Pakistan; GIH: Gujarati Indians in Houston, Texas, USA; JPT: Japanese in Tokyo, Japan; LWK: Luhya people in Webuye, Kenya; MXL: Mexican Ancestry in Los Angeles, Colombia; PEL: Peruvian in Lima, Peru; TSI: Toscans in Italy; YRI: Yoruba in Ibadan, Nigeria.
